# Edge detection algorithm of cancer image based on deep learning

**DOI:** 10.1080/21655979.2020.1778913

**Published:** 2020-06-21

**Authors:** Xiaofeng Li, Hongshuang Jiao, Yanwei Wang

**Affiliations:** a Department of Information Engineering, Heilongjiang International University, Harbin, China; b Office of Academic Research, Heilongjiang International University, Harbin, China; c Department of Mechanical Engineering, Purdue University, Indianan, IN, USA

**Keywords:** Deep learning, cancer imaging, edge detection, feature extraction, three-dimensional reconstruction

## Abstract

For the existing medical image edge detection algorithm image reconstruction accuracy is not high, the fitness of optimization coefficient is low, resulting in the detection results of low information recall, poor smoothness and low detection accuracy, we proposes an edge detection algorithm of cancer image based on deep learning. Firstly, the three-dimensional surface structure reconstruction model of cancer image was constructed. Secondly, the edge contour feature extraction method was used to extract the fine-grained features of cancer cells in the cancer image. Finally, the multi-dimensional pixel feature distributed recombination model of cancer image was constructed, and the fine-grained feature segmentation method was adopted to realize regional fusion and information recombination, and the ultra-fine particle feature was extracted. The adaptive optimization of edge detection was realized by combining with deep learning algorithm. The adaptive optimization in the process of edge detection was realized by combining with the deep learning algorithm. The experimental results show that the three-dimensional reconstruction accuracy of the proposed algorithm is about 95%, the fitness of the optimization coefficient is high, the algorithm has a strong edge information detection ability, and the output result smoothness and the accuracy of edge feature detection are high, which can effectively realize the detection of cancer image edge.

## GRAPHICAL ABSTRACT

**Figure:**
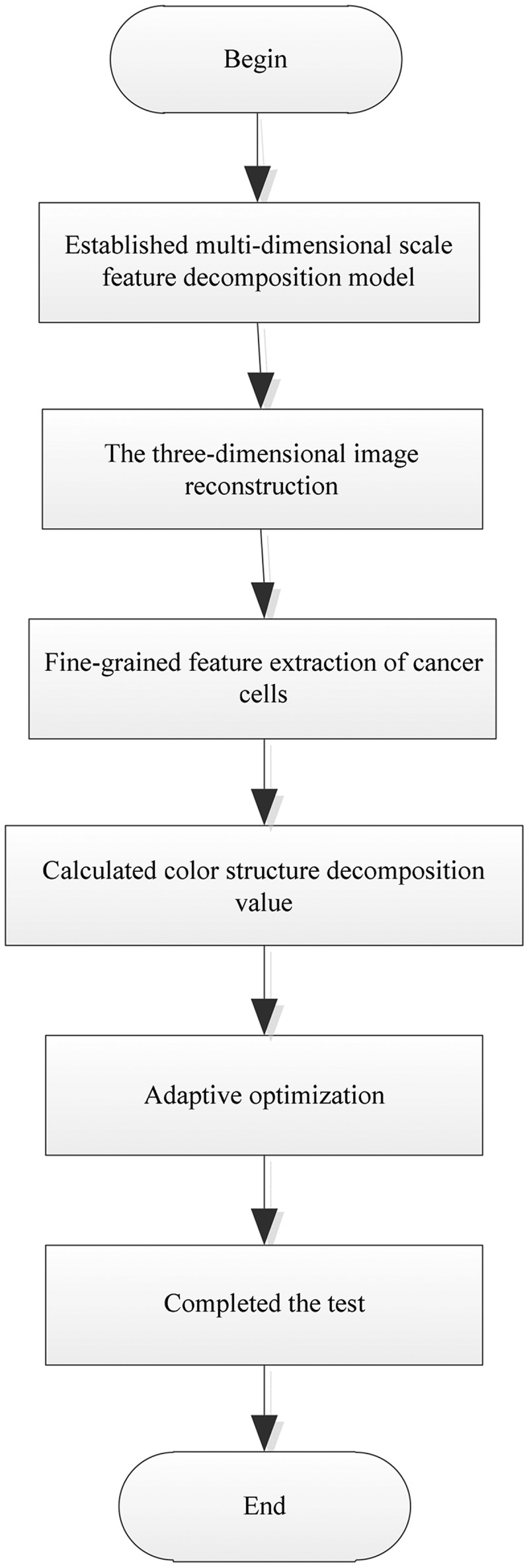

## 1. Introduction

With the development of imaging technology, the use of medical imaging methods for pathological detection has become one of the important means of medical treatment while research on medical image detection methods is the basis of medical diagnosis [1]. Through the edge contour detection and segmentation on the cancer image, the three-dimensional feature quantity of the cancer image can be extracted, and the three-dimensional detection of the cancer image can be realized. And reconstructing the three-dimensional feature quantity of the cancer image can improve the ability of adaptive detection and feature recognition of the cancer image. Therefore, related researches on cancer image detection methods have received great attention [2,3].

Deep learning is a branch direction in the field of machine learning. It is usually based on artificial neural networks to perform representation learning on data [4,5]. Deep learning methods can give computer equipment the ability to train and learn images and data. At present, deep learning has been widely used in data mining, information retrieval, natural language processing, personalized services and other fields [6], especially in medical image analysis.

In literature [7], the convolution neural network in depth learning is used for image sorting research. The image is input into the convolution neural network, the hierarchical features of the image are extracted through supervised learning, and the features are normalized. Then the image retrieved is sorted by manifold ranking method, and the image retrieval is completed efficiently. In literature [8], Image Emotion Feature Extraction Based on convolution neural network model based on deep learning, the image low-level features and high-level emotions are integrated, and the convolution neural network model is improved. The improved model is used to calculate image information, and the back propagation algorithm is used to verify the accuracy of the calculation model processing results, and good results are obtained. Literature [9] proposed that deep learning algorithms such as convolutional neural networks can automatically extract hidden disease diagnosis features from medical image data. It is currently being used to analyze medical images. This paper analyzed the principles of deep learning and focuses on convolution neural network, summarized the framework of image classification and segmentation, described the latest progress of deep learning-based medical image analysis methods. Literature [10] proposed that accurate and reliable brain tumor segmentation is a key component of cancer diagnosis, treatment planning, and evaluation of treatment results. This paper integrated fully convolutional neural networks and conditional random fields into a unified framework based on deep learning technology, developed a brain tumor segmentation method and obtained the segmentation results. Literature [11] proposed several deep learning evaluation methods for automated bone and bone age, which greatly improved the shortcomings of traditional methods and gave full play to the advantages of deep learning. Literature [12] combined deep learning ideas and iterative quantization ideas, conducted image retrieval research based on convolutional neural networks and iterative quantization, trained neural network models, extracted image features, and used the principle of minimum error between image feature values and hash code values, selected the optimal value for image retrieval, the results show that this method is superior to mainstream retrieval algorithms.

On the basis of the existing research results of deep learning, this paper proposes a cancer image edge detection algorithm based on deep learning. In order to provide more data analysis basis for cancer research, the edge detection is completed by the reconstruction of cancer image. The algorithm first performed three-dimensional reconstruction of cancer images and extracted fine-grained features of cancer cells. On this basis, it decomposed the color structure features of cancer images, and used deep learning technology to detect and complete the edge information of cancer images. The experimental results show that the performance of the proposed algorithm is superior, which provides a certain reference for the future development of medical field. The main contributions of this paper are as follows:

1. The reconstruction model of three-dimensional surface structure of cancer image provides the basis for edge detection of cancer image;
2. Multi-scale color structure feature decomposition of the image to build a multi-dimensional pixel feature distributed recombination model;
3. The edge detection process of cancer image is optimized by using deep learning algorithm, and the edge detection of cancer image is realized.
4. The accuracy of three-dimensional reconstruction of cancer image, the fitness of optimization coefficient, the recall, smoothness and accuracy of edge detection results are used to verify the analysis, which increases the reliability of experimental results.

## 2. Related work

Detection of cancer image edges is the basis for cancer diagnosis and treatment. At present, some experts and scholars have proposed some mature research results in this field. For example, cancer image detection method based on wavelet transform, cancer image edge detection method based on feature segmentation, statistical feature component method, and fuzzy edge detection method [13]. These methods are based on the establishment of an image information fusion model for edge detection of cancer image, and use dynamic fusion methods for cancer image detection. In addition, in Literature [14], an image contour detection algorithm based on the association of multiple receptive field orientations of the visual pathway is proposed. On the basis of constructing a neural network that detects spatially different information, the algorithm encodes the contour information of the image beforehand, and then establishes a non-classical receptive field adjustment mechanism. Adjust the pre-stage coding according to its hierarchical transformation characteristics. Besides, a multi-receptive field orientation correlation model is proposed to suppress and threshold contour responses in different directions to achieve contour detection. Literature [15] proposed a block adaptive color image edge detection method based on Convolutional Neural Networks (CNN). To ensure the accuracy of edge detection results and effectively suppress noise, this method performs adaptive block detection on the image, and uses the entropy function to measure the properties of each sub-region in the image. Based on the measurement results, appropriate network parameters are selected, the CNN template for image edge extraction is analyzed, and the CNN robustness theorem is designed to ensure the stability of image edge process detection. However, the above two methods have weak ability to distinguish features and poor self-adaptation due to environmental influences when performing edge detection of cancer image. In Literature [16] proposes an image corner detection algorithm. This paper uses Canny edge detector to extract the edge contour line, and smoothes the edge line. The point chord distance recursive calculation method is used to select the point chord distance by repeated calculation Optimal candidate corner points, and finally multi-scale technology is used to determine the candidate corner point and get the final corner point, and a better detection result is obtained. However, the accuracy still needs to be improved. Literature [17] proposed an image edge detection algorithm based on sparse representation. This algorithm first cuts the image and obtains its sparse representation results. In the morphological edge detector, iterative processing and noise filtering step direction sub-band coefficients. On this basis, the edges of each subband are combined using Dempster-Shafer theory, and the complete image edge detection information is obtained through continuous iteration. However, the above two algorithms are difficult to effectively restrain the pulse interference caused by noise, resulting in poor smoothness of the output result. Literature [18] proposed a medical image edge extraction algorithm based on Sobel operator. This algorithm is suitable for CT and MRI equipment. Sobel operators are programmed into the TMS320DM6446 chip by DSP programming, and the edge features of medical images are quickly detected and extracted according to the operating principle of the Sobel algorithm. However, the accuracy of the algorithm for edge information detection is low.

Therefore, in order to address the problems in the traditional methods, this paper designs a cancer image edge detection algorithm based on deep learning. Before the edge detection of cancer images, a three-dimensional reconstruction analysis of cancer images was first performed, which provided a basis for improving the accuracy of the detection results. The experimental results show that the accuracy of three-dimensional reconstruction of cancer image is up to 95%, the fitness of optimization coefficient is high, the precision of detection is up to 95%, the smoothness is good, and the accuracy of edge information detection is high.

## 3. Cancer image reconstruction and extraction of fine-grained features of cancer cells

### 3.1. The three-dimensional reconstruction of cancer image

In order to realize the edge detection of cancer image based on deep learning, the ability of image detection is improved. It is necessary to first perform three-dimensional reconstruction of cancer image, and perform feature extraction based on the three-dimensional reconstruction results of cancer image [19,20]. Therefore, a three-dimensional feature reconstruction model of a cancer image is constructed, and a combination of edge contour feature extraction methods is used to extract fine-grained features of cancer cells from the cancer image. Multidimensional feature segmentation is required for cancer image. And combined with fuzzy information fusion technology, multi-dimensional decomposition in the process of cancer image reconstruction is performed to improve the feature analysis ability of cancer image reconstruction [21,22].

**Figure 1.**
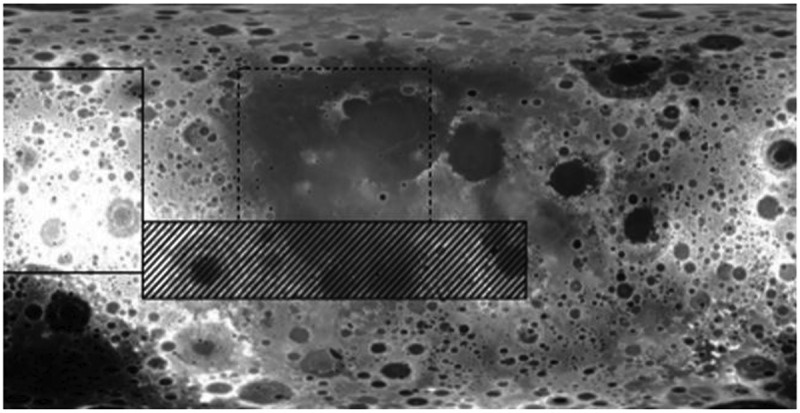
Edge contour feature detection model of cancer image.

In the affine invariant region and Cartesian space, a multi-dimensional feature distribution model of cancer image is constructed, and multi-dimensional feature recognition and information fusion methods are used for edge detection and fine-grained feature decomposition of cancer image. An edge contour feature detection model of a cancer image is obtained according to the above process. It is shown in Figure 1.

Combine the edge contour feature detection model of the cancer image in Figure 1 to reconstruct the edge contour of the cancer image to construct a linear fusion model of the cancer image. Contour feature distribution areas at the edges of cancer image. Suppose$A=\{a_{m}{\}}_{m=1}^{n}$is the cancer image scalar pixel sequence composed byn scalars. The multi-scale fusion method is used to perform the color difference calibration of the cancer image template construction [23,24]. The multi-dimensional feature decomposition model meets the following constraints:

(1) $$\underset{P\to -\infty }{\lim}\kappa A^{P}=\underset{m}{\min}a_{m}$$

where, κis the spatial dimension. P represents the sequence vector of cancer image pixels. $a_{m}$represents the scalar image sequence of cancer image. mrepresents the maximum number of subsequences.

**Figure 2.**
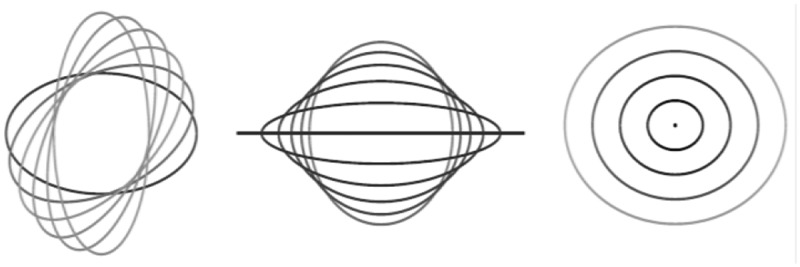
Three-dimensional reconstruction process of cancer image.

Based on this, the adaptive pixel-limited reconstruction method is used to reconstruct the gray pixel features during the detection of edge contour features of cancer image. The gray pixels of the cancer image are$\left(x,y\right)$. The results of cancer image cell types with different attribute sets are used to directly reconstruct cancer image in three-dimensional. It is shown in Figure 2.

According to the three-dimensional contour reconstruction results, combined with the ambiguity feature decomposition method, multi-scale color structure feature decomposition is performed on cancer image to construct a multi-dimensional pixel feature distributed recombination model of cancer image. This can improve the three-dimensional feature reconstruction capabilities of cancer image.

### 3.2. Extraction of fine-grained features of cancer cells

On the basis of the above three-dimensional reconstruction of cancer image, the edge contour feature extraction method is used to extract fine-grained features of cancer cells of cancer image, and the matching points on the cancer image grid model are calculated [25,26]. Design the multi-component transfer function expression of the cancer image:

(2) $$g=\left\{\begin{array}{c} Rs_{j},z\leq i\leq x-y\\ g_{i},\;otherwise \end{array}\right.$$

where, iis set of edge pixels for cancer image, Rrefers to a canonical constant, $s_{j}$refers to the cancer image blur factor, and $g_{i}$refers to the two-dimensional contour feature matching coefficient of cancer image. Multi-component pixel spatial feature reconstruction and quantization decomposition methods are used to perform RGB decomposition of cancer image. In the i RGB feature component, get the grayscale pixel level of the cancer image$g_{i}$, and calculate the characteristics of cancer cell distribution in cancer image. Perform block matching of cancer image images according to the shape of the template. The pixel set intensity of the cancer image is:

(3) $$N=\frac{\mu \times g}{{\left(c_{0}\right)}^{\gamma }}$$

where, μis the center moment of cancer image, $c_{0}$refers to the zero-order moments for fine-grained feature extraction of cancer cells in cancer imaging, and γrefers to the order. Color template feature matching method is used to detect edge contour features of cancer image. At the center pixel of the map distributionv, the center moment of the edge detection of the cancer image is $\left(x_{v},y_{v}\right)$. For the two-dimensional contour feature distribution point *M*, the edge contour feature distribution function of the mesh model in which it is located is: Eis

(4) $$E=s\left(\delta^{2}-\frac{df(x)}{dx}\right)+\beta N$$

where, sis the tone mapped edge scale, and $\delta^{2}$is the local variance of cancer imaging images, and$f\left(x\right)$is the response function, $\beta =\max\left(\frac{\delta^{2}-f(x)}{\delta^{2}}\right)$ represents the maximum value of edge contour eigenvector. The two-dimensional reconstruction of the cancer image is performed in a high dynamic range, and the gray values of the pixels at different frames of the cancer image are obtained through the image synthesis and tone mapping [27] processing to achieve fine-grained feature extraction of cancer cells.

## 4. Edge detection algorithm for cancer image based on deep learning

### 4.1. Color structure feature decomposition of cancer image

Based on the above-mentioned edge contour feature extraction method for fine-grained feature extraction of cancer cells of a cancer image, edge detection of the cancer image is performed. Deep learning technology can be used to effectively mine the underlying structure of high-dimensional data, so an edge detection algorithm for cancer image based on deep learning is proposed. The pixel feature is optimized according to the color space block fusion result of the cancer image [28], and the multi-scale color structure feature decomposition is performed on the cancer image in combination with the ambiguity feature decomposition method, and the feature decomposition output is:

(5) $$T=\frac{kE}{\sqrt{\frac{1}{m}\times u_{j}(k)}}$$

where, kis cancer image density of cancer image, *m* refers to feature decomposition scale of cancer image, $u_{j}(k)$refers to the cancer pixel edge pixel intensity, and jrefers to the maximum feature distribution density of cancer image.

**Figure 3.**
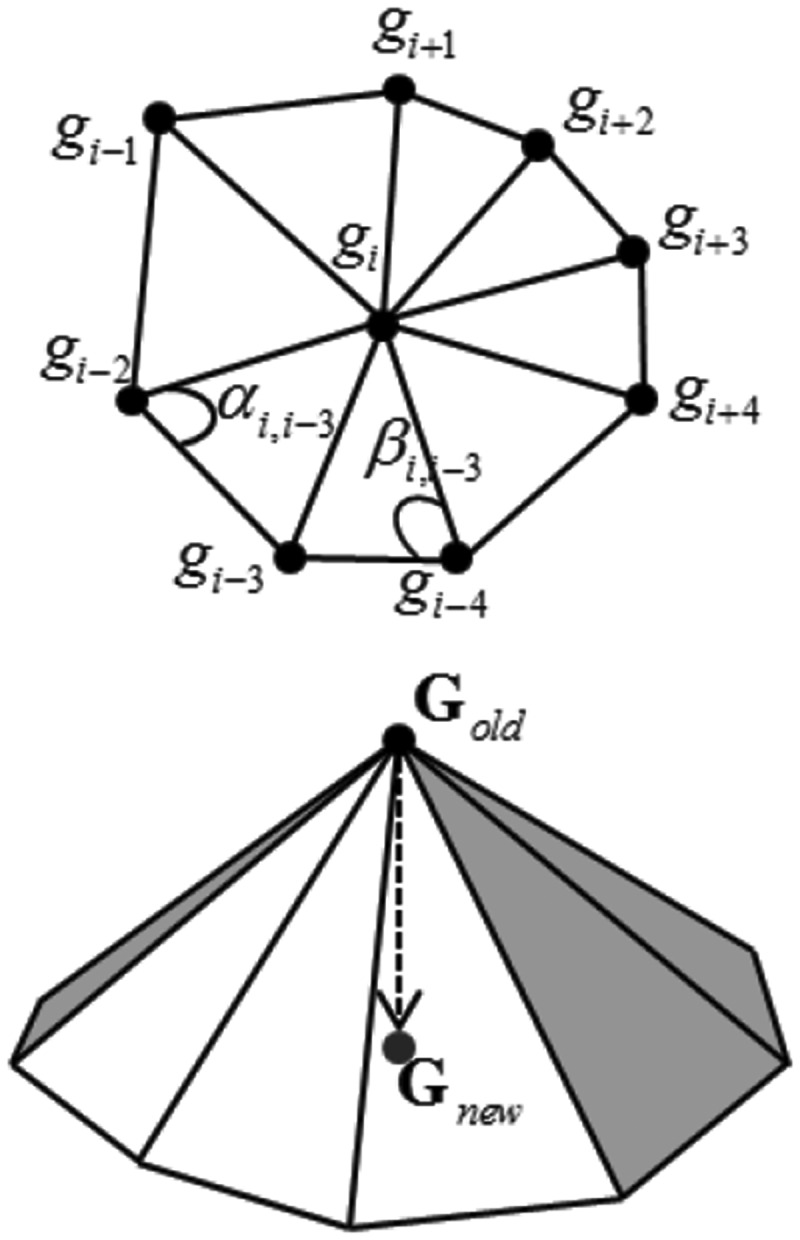
Reconstruction results of edge contour features of cancer image.

On this basis, multi-dimensional feature decomposition and information recombination are performed on the cancer image to reconstruct the edge contour feature distribution set of the cancer image, that is, the image content of each region. The feature amount is extracted through the pre-trained CNN, and then the true contour corresponding to the feature amount in the theoretical dictionary is found by the nearest neighbor search algorithm, and the edge contour feature reconstruction is realized through the contour output. It is shown in Figure 3.

In the reconstruction result of the edge contour features of the cancer image in Figure 3, the pixel features of the cancer image are reconstructed in combination with the detail distribution.

(6) o(x,y)=b(x,y)+ε(x,y)

where, o(x,y), b(x,y)and ε(x,y) represent the original image output results, binary image output results, and grayscale image output results of cancer image images . Use the variance fusion method to perform window matching of cancer image, the fuzzy correlation coefficient isη. During the reconstruction of the cancer image, an edge contour feature detection model for the cancer image is constructed, and the output edge contour feature detection function is: Fis

(7) $$F=\frac{\eta T}{o\left(x,y\right)}$$

Based on this, combined with the active contour detection method, the color structure feature decomposition value and the color structure feature output value of the cancer image are obtained:

(8) $$C=\partial \left(F+\left(1-\partial \right)T\right)$$

where, ∂ represents the correlation coefficient of the cancer image’s pixel matching window. Then, fine-grained feature segmentation is used to achieve regional fusion and information recombination of cancer image, and perform edge detection.

### 4.2. Establishment of a deep learning-based detection algorithm

The medical image is divided into blocks of the same size with the pixel as the center. The features in the blocks are extracted by the neural network model after pre training, and the extracted features are compared with the real contour to obtain the contour probability of the corresponding pixels in the blocks. Combine contour probabilities for adaptive optimization in edge detection of cancer image, and the deep learning process of cancer image is described as:

(9) $$L=C\times \left(S+z\right)$$

where, S represents the number of iteration steps for edge detection of cancer image. zrefers to the number of partitions in deep learning. Suppose the grayscale pixel subset of cancer image isV. The weighted average method is used to jointly detect the color and texture of the image, and the statistical distribution function of the obtained cancer image is described byI. Divide the cancer image into p×q2×2 sub-blocks$I_{pq}$. The wavelet multi-scale decomposition method was used to perform binary reconstruction of cancer image. The low dynamic range detection sequence output of cancer image is:

(10) $$H=rI_{pq}\left(\omega +L/2\right)$$

where, ωis the gray pixel values of cancer image images in joint affine invariant area, andrrefers to the order of the spatial location of cancer image. Through the above algorithm design, edge contour detection of cancer image is performed, and deep learning algorithm is used to perform adaptive optimization in the edge detection process of cancer image. Assume that the optimization coefficient in the deep learning process isϕ, the edge contour detection output is:

(11) $$Z=\theta H{\left[\frac{\left(\lambda_{2}-\lambda_{1}\right)}{V}\right]}^{\phi }$$

where, θis feature value of output component of cancer image, and$\lambda_{1},\lambda_{2}$ are the long and short semi-axis lengths in the deep learning optimization process.

### 4.3. The proposed algorithm

According to the above research, the color structure features of cancer image are extracted, and the detection algorithm is established. Based on this, the ultra-fine-grained features of cancer image are further extracted, and finally the edge detection of cancer image is completed efficiently. The specific steps are as follows:

Input: Color structure features and fine-grained features of cancer images;

Output: Edge detection results of cancer images.

Initialized the original data of the cancer image to detect the edge of the cancer image. The specific algorithm is described as:

(1) Use cancer image scalar pixel sequence A and multi-scale fusion method to obtain multi-dimensional feature decomposition model;

(2) The adaptive parameter limited reconstruction method is used to realize the image gray pixel feature recombination to obtain the gray pixel points$\left(x,y\right)$ of cancer image.

(3) Calculate the edge contour feature distribution function E according to the multi-component transfer function g of the cancer image and the color template feature matching process;

(4) The two-dimensional reconstruction of the video image in a high dynamic range, to obtain the pixel gray values at different frames, and to extract the fine-grained characteristics of cancer cells;

(5) Combine the ambiguity feature decomposition method to obtain the color structure feature output valueC;

(6) Construct a multi-dimensional pixel feature distributed recombination model of cancer image according to the feature output valueC, and extract the ultra-fine-grained feature amount in the fine-grained feature segmentation process

(7) Combine the deep learning process L to obtain the contour probability of the pixels corresponding to the segmented block, and use the optimization coefficient ϕ to detect and output the edge contour.

(8) End

**Figure 4.**
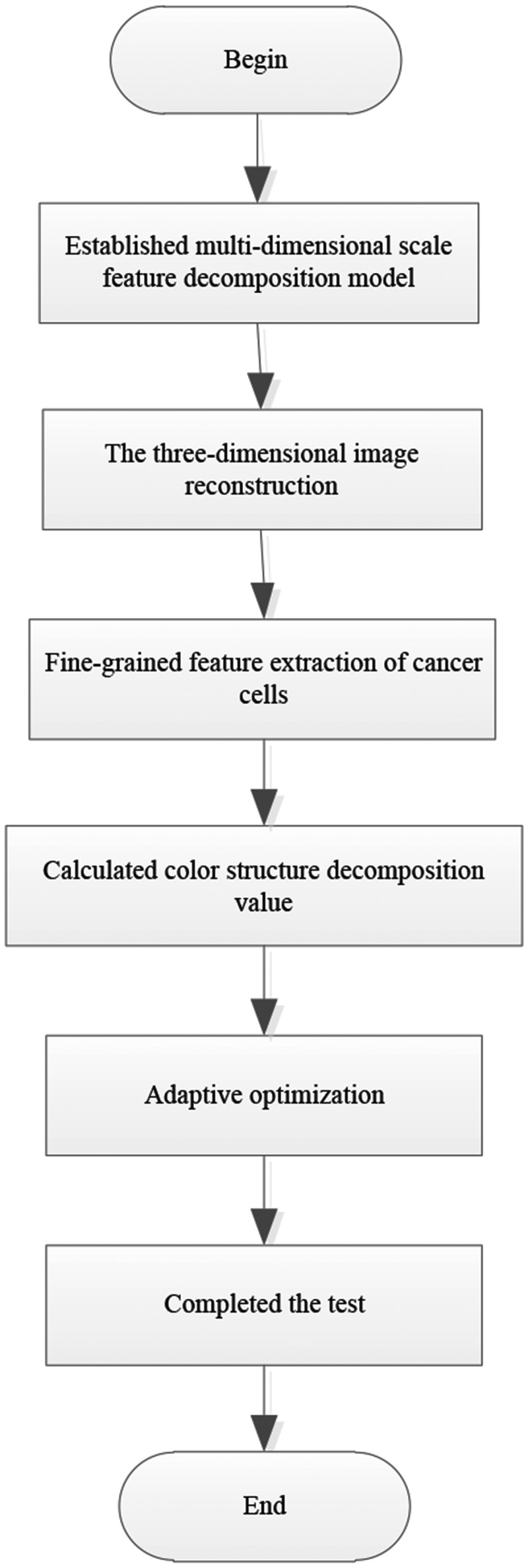
Cancer image edge detection flow.

Combined the above steps to complete the detection of the edge of the cancer image. It is shown in Figure 4.

## 5. Experiments and results analysis

### 5.1. Experimental environment and data sets

The experimental environment of this paper is Windows10 system, Intel i5 dual core CPU3.40 GHz, 4GB DDR3 memory. The experimental environment was performed on the Matlab platform, and the data came from the COSMIC data sets (https://cancer.sanger.ac.uk/cosmic/). Selected a total of 4 data sets from CNA, methylation, gene fusion and SNP in the COSMIC dataset. The data sets are described as follows:

According to the Table 1, the number of image test samples in this experiment is 4 million, and the number of iterations for cancer image acquisition is 500. The initial learning rate for cancer image detection is 0.12, and the attenuation weight coefficient is 0.001.

**Table 1.** Experimental data sets.

Data sets	Number of samples/ten thousand	Training data/ten thousand	Test data/ten thousand
CAN	300	200	100
Methylation	300	200	100
Gene fusion	300	200	100
SNP	300	200	100

### 5.2 Experimental steps

1. Selected the deep learning framework tensorflow to input the experimental sample data, built and train the neural network model;

2. Based on neural network model, cancer image reconstruction and edge detection were processed;

3. Outputed cancer image edge detection results;

4. Compared the experimental results of proposed algorithm with the results of traditional algorithms to verify the performance of proposed algorithm. Selected the literature [4], literature [6], literature [11] and literature [12] algorithms for comparative analysis

### 5.3 Experimental indexs

1. The three-dimensional reconstruction accuracy of cancer images

The three-dimensional reconstruction analysis of cancer images provided a good basis for edge detection. In order to verify the performance of the proposed algorithm, the proposed algorithm was compared with the three-dimensional reconstruction accuracy of the algorithms in literature [4], literature [6], literature [11] and literature [12].

1. Fitness of optimization coefficient ϕ

The fitness of optimization coefficient ϕ has a significant impact on the effectiveness of the final detection results. Therefore, the fitness of optimization coefficientϕ is selected as the index for verification and analysis.

1. Recall rate

The recall rate refers to the ratio of the amount of relevant edge information extracted to the total amount of edge information extracted by the edge detection process, and is usually determined by the amount of edge information and the stability of the extraction environment. It is an index to measure the adaptive ability of the detection algorithm, and its calculation process is as follows:

(12) $$Recall\;rate=\frac{Amount\;of\;extracted\;edge\;information}{Total\;amount\;of\;edge\;information}\times 100\%$$

1. Smoothness of output results

The essence of the edge detection process is a filtering process, and the smoothness of its output can reflect the anti-interference ability and stability of the detection algorithm. To this end, the W consecutive extracted values of the edge information are regarded as one echelon, and each newly sampled data is placed at the end of the line once. At the same time, the data of the corresponding position at the head of the team is discarded. An arithmetic average operation is performed on the W data to obtain the final filtering result, and the high frequency oscillation range and smoothness are observed.

1. Accuracy of edge information detection

This indicator can be used to judge the correctness and reliability of different detection algorithms in edge feature detection and extraction. The calculation process is as follows:

(13) $$Accuracy=\frac{Correct\;number\;of\;edge\;detections}{Total\;number\;of\;edge\text{\textbackslash{}detections}}\times 100\%$$

### 5.4. Rusults and discussion

**Figure 5.**
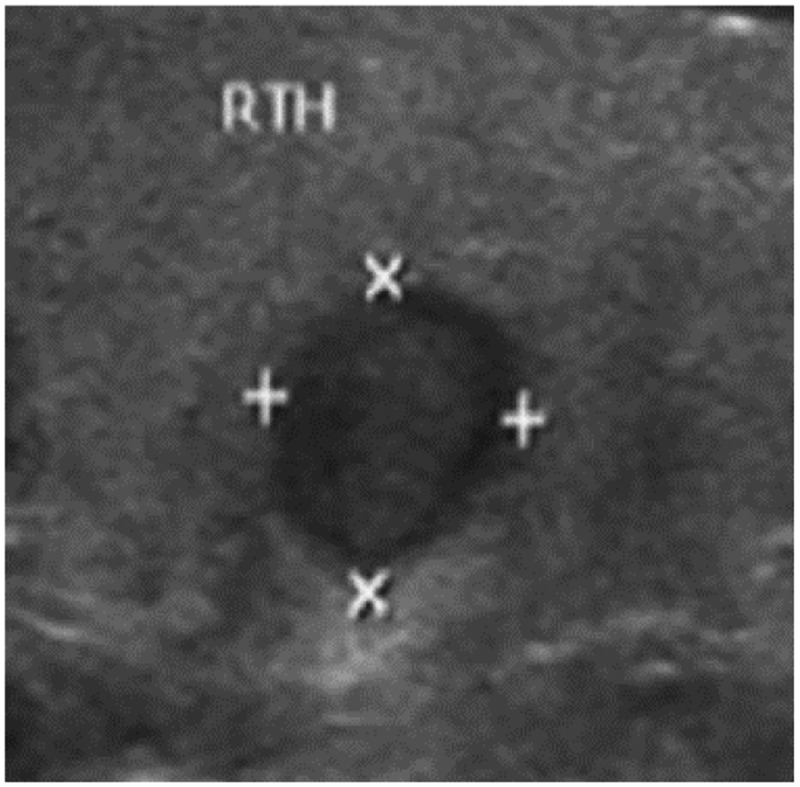
Original cancer image.

According to the above experimental environment and the setting of indicators, edge detection of cancer image is performed. Take the thyroid cancer ultrasound image as an example for edge detection. First, the cancer image data is collected to obtain the original image. It is shown in Figure 5.

**Figure 6.**
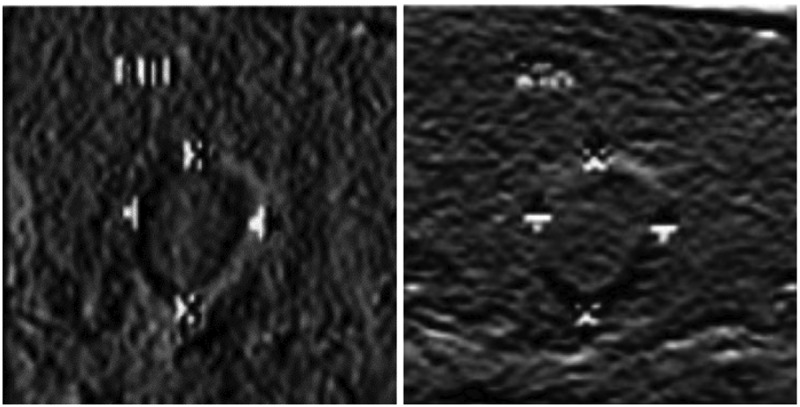
Fusion results of fine-grained features of cancer image.

The original cancer image in Figure 5 is used as the research sample, and 548 thyroid ultrasound images are selected as the test sample set for cancer screening. A three-dimensional surface structure reconstruction model of cancer image was constructed, and multi-scale color structure feature decomposition and detection were performed on the cancer image in combination with the ambiguity feature decomposition method to obtain fine-grained feature fusion results. It is shown in Figure 6.

**Figure 7.**
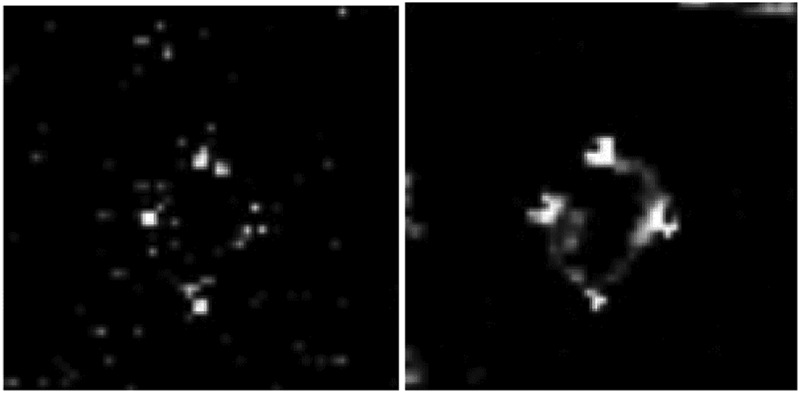
Edge detection output of cancer image.

It can be seen from Figure 6 that the fine-grained features of the cancer image are more obvious, and the edges show a plurality of arcs with obvious unevenness. This proves that the proposed algorithm can effectively achieve fine-grained feature detection and information fusion of cancer cells in cancer image. On this basis, edge detection is performed on cancer image. It is shown in Figure 7.

It can be seen from Figure 7 that the edge information of the cancer image is obvious and the nodule shadow is clear. This proves that the self-adaptive convergence of cancer image edge detection using the cancer image edge detection algorithm based on deep learning is better, the feature resolution capability is stronger, and the cancer cell recognition and resolution capability is better.

In order to further test the effectiveness of the deep learning-based edge detection algorithm for cancer image, a comparative experiment is designed as follows.

1. Comparison of the three-dimensional reconstruction accuracy

**Figure 8.**
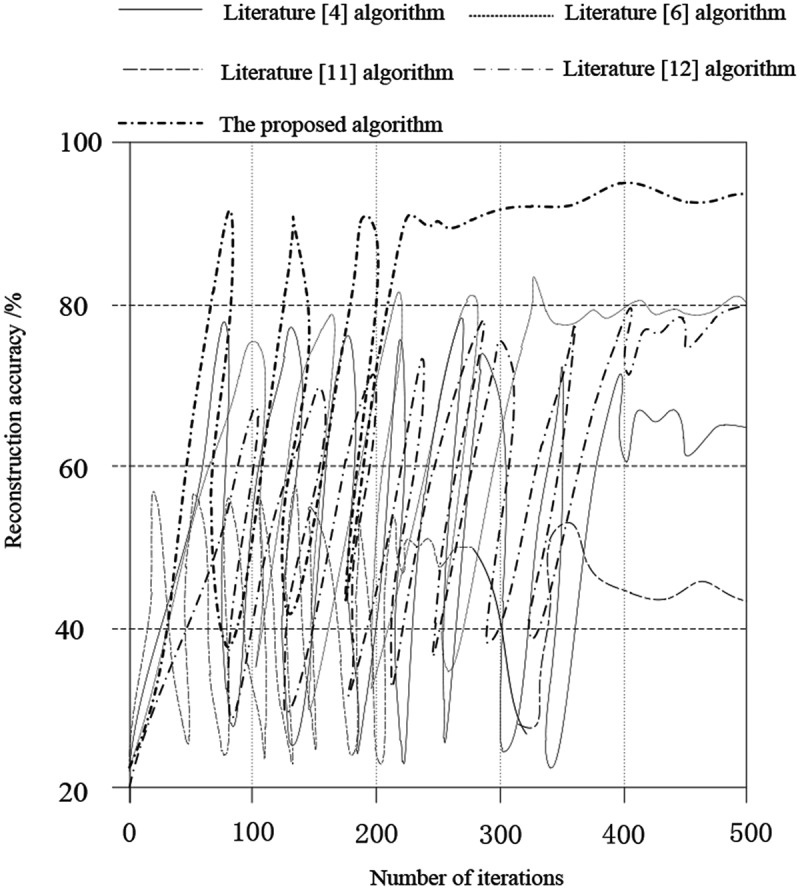
Comparison of the three-dimensional reconstruction accuracy.

Comparing the proposed algorithm with the algorithms of literature [4], literature [6], literature [11] and literature [12], the accuracy of the three-dimensional reconstruction of cancer images is shown in Figure 8.

According to Figure 8, in the iterative process, the accuracy of the three-dimensional reconstruction of cancer images of the proposed algorithm is always higher than that of other literature algorithms. When the number of iterations is 500, the accuracy of the three-dimensional reconstruction of the proposed algorithm is as high as about 95%. The highest accuracy of the algorithm in literature [6] and literature [12] can reach 80%. The highest accuracy of the algorithm in literature [4] is about 65%. Literature [11] has the lowest algorithm and the highest accuracy can only reach about 45%. According to the above data analysis, we can find that the proposed algorithm has some advantages, because the proposed algorithm constructs a multi-dimensional scale feature decomposition model, and completes image reconstruction based on this, and the reconstruction accuracy is high.

1. Comparison of fitness of optimization coefficient ϕ

The fitness of the optimization coefficient is selected as the index, and the percentage value is used to represent the calculation results of different algorithms, as shown in Table 2.

**Table 2.** Fitness of optimization coefficientϕ.

Algorithm	ϕ fitness/%
Literature [4] algorithm	75.3
Literature [6] algorithm	65.2
Literature [11] algorithm	69.8
Literature [12] algorithm	70.5
The proposed algorithm	90.6

According to the analysis of Table 2, the adaptability of the optimization coefficientϕ of the proposed algorithm is as high as 90.6%, and the coefficientϕ of the algorithm in the literature [6] is the lowest, only 65.2%. The algorithm in Literature [4] is relatively high, but it is still 15.3% lower than the proposed algorithm. It can be seen that the optimization coefficient ϕ selected by the proposed algorithm is more suitable, which is helpful to improve the accuracy of edge detection.

1. Comparison of recall rate

**Figure 9.**
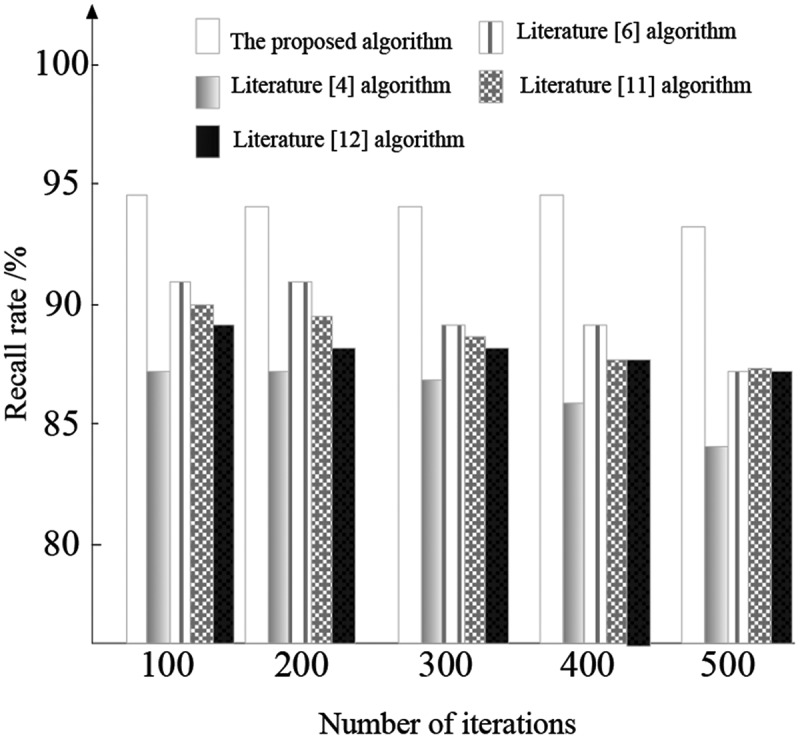
Comparison of recall rate of different image edge detection algorithms.

The edge information recall rate of cancer image detection is tested by different algorithms, and the result is shown in Figure 9.

It can be seen from Figure 9 that with the continuous increase of the number of experimental iterations, the edge information recall rate of different image edge detection algorithms also changes continuously, and the overall performance shows a downward trend. However, the edge information recall rate of the proposed cancer edge detection algorithm based on deep learning has always maintained the highest of the five methods, approaching 95%. This proves that the algorithm has strong self-adaptability and strong ability to distinguish edge features.

1. Comparison of smoothness of output results

**Figure 10.**
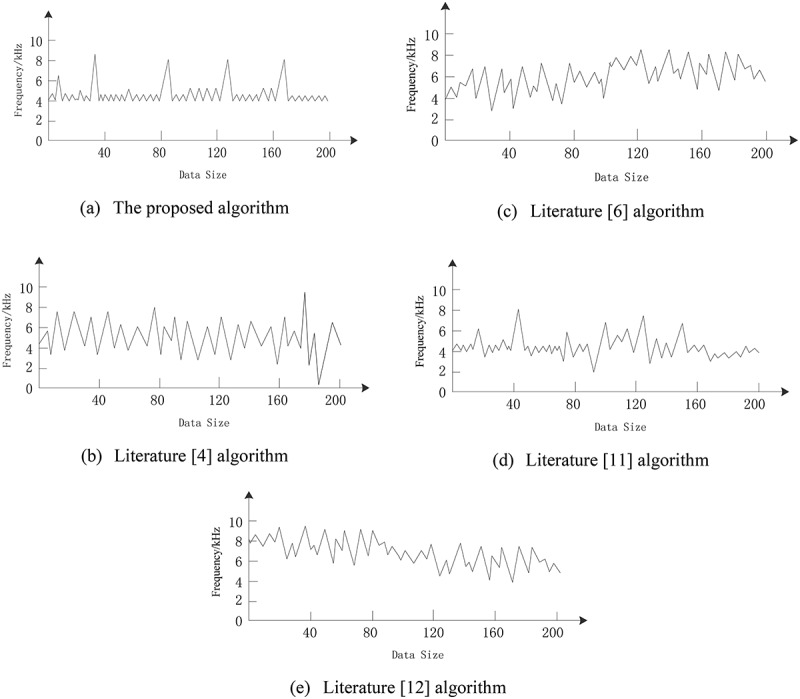
Comparison of output smoothness of different image edge detection algorithms.

The smoothness of the output results of cancer image detection is tested by different algorithms, and the result is shown in Figure 10.

It can be seen from Figure 10 that with the continuous increase in the amount of data, the output frequency of different image edge detection algorithms also constantly changes. The output frequency of the deep learning edge detection algorithm for cancer image is relatively regular and the fluctuations are stable, which can explain that the edge detection output results are smoother under this algorithm, which proves that the algorithm has a high degree of smoothness.

1. Comparison of a ccuracy of edge information detection

Comparing the accuracy of edge information detection with different algorithms, the results are shown in Table 3.

**Table 3.** Comparison of detection accuracy of different image edge detection methods (%).

Sample size/ten thousand	The proposed algorithm	Literature [4] algorithm	Literature [6] algorithm	Literature [11] algorithm	Literature [12] algorithm
100	88.1	80.5	83.2	80.1	83.4
200	87.9	79.5	78.4	78.5	85.6
300	87.5	82.1	75.3	78.1	86.0
400	86.1	78.1	81.6	77.3	81.4

It can be seen from Table 3 that with the continuous increase of the number of experimental samples, the detection accuracy of different image edge detection methods is constantly changing, but there is no obvious change. The detection accuracy of the algorithms in Literature [4], Literature [6] and Literature [11] is obviously smaller than that of the proposed algorithm. The detection accuracy of the algorithm in Literature [12] is close to that of the proposed algorithm, but still smaller than the proposed algorithm. It is proved that the proposed cancer edge detection algorithm based on deep learning has higher accuracy and less error in edge feature detection, and has stronger ability to distinguish and recognize edge features of cancer image.

## 6. Conclusions

Through the edge contour detection and segmentation on cancer image, three-dimensional detection of cancer image can be completed. Therefore, we proposes an edge detection algorithm for cancer image based on deep learning. Construct a linear fusion model of cancer image, use color template feature matching methods to detect edge contour features of cancer image, perform two-dimensional reconstruction of cancer image images in a high dynamic range, and use deep learning algorithms to detect edges of cancer image. It is known that the proposed algorithm has higher three-dimensional image reconstruction accuracy and optimization coefficient selection. The proposed algorithm has higher accuracy for cancer image detection, better feature resolving ability, and stronger practicability. However, the application range of this algorithm needs to be further improved. In future research, the algorithm will continue to be optimized, so that the optimized algorithm can be applied to the detection of more types of medical images.
